# Development and evolution of caste dimorphism in honeybees – a modeling approach

**DOI:** 10.1002/ece3.414

**Published:** 2012-11-08

**Authors:** Olof Leimar, Klaus Hartfelder, Manfred D Laubichler, Robert E Page

**Affiliations:** 1 Department of Zoology, Stockholm UniversityStockholm, Sweden; 2 Wissenschaftskolleg zu BerlinWallotstrasse 19, Berlin, Germany; 3 Departamento de Biologia Celular e Molecular e Bioagentes Patogênicos, Universidade de São PauloRibeirão Preto, São Paulo, Brazil; 4 School of Life Sciences, Arizona State UniversityTempe, Arizona

**Keywords:** Caste determination, developmental evolution, plasticity, polyphenism, social insects

## Abstract

The difference in phenotypes of queens and workers is a hallmark of the highly eusocial insects. The caste dimorphism is often described as a switch-controlled polyphenism, in which environmental conditions decide an individual's caste. Using theoretical modeling and empirical data from honeybees, we show that there is no discrete larval developmental switch. Instead, a combination of larval developmental plasticity and nurse worker feeding behavior make up a colony-level social and physiological system that regulates development and produces the caste dimorphism. Discrete queen and worker phenotypes are the result of discrete feeding regimes imposed by nurses, whereas a range of experimental feeding regimes produces a continuous range of phenotypes. Worker ovariole numbers are reduced through feeding-regime-mediated reduction in juvenile hormone titers, involving reduced sugar in the larval food. Based on the mechanisms identified in our analysis, we propose a scenario of the evolutionary history of honeybee development and feeding regimes.

## Introduction

**Figure 1:**
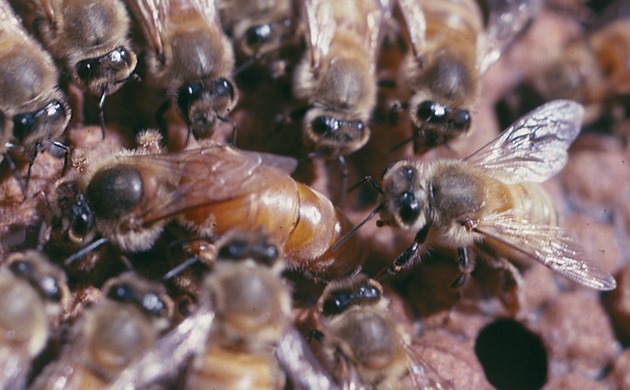
A honeybee queen (center left) attended by her retinue of workers. Photo by Harry H. Laidlaw Jr.

Eusocial insects are characterized by a reproductive division of labor and overlapping generations (Michener 1969; Wilson 1971; Hölldobler and Wilson 2009). In the highly eusocial insects, there is a queen–worker caste dimorphism, with morphologically and physiologically distinct reproductive queens and more or less sterile workers, which involves a division of labor that includes brood care. A honeybee queen may lay up to 2000 eggs per day during the spring, whereas workers normally only lay eggs in the absence of the queen and young larvae. Queens and workers display strong diphenism where workers have a much lower body mass than queens (Fig. 1; Linksvayer et al. 2011), have two small ovaries containing few ovarioles, a vestigial spermatheca, a barbed sting used in defense of the nest, and mid and hind leg structures adapted for pollen collection and transport. Queens, on the other hand, have two large ovaries that contain many more ovarioles. In addition, the queen has a shorter tongue, nonbarbed sting, and lacks the pollen collection structures on the legs.

A major concern for the study of social insects is to explain how the caste dimorphism evolved. This dimorphism is a well-studied and intriguing case of developmental plasticity and polyphenism, which throws light on such basic issues as whether plasticity is a continuous reaction norm or a discontinuous switching between phenotypes (Nijhout 2003). It has the striking property that socially determined environmental circumstance plays a role in inducing the dimorphic development, for instance through the feeding behavior of nurse workers. In this sense, the emergence of caste dimorphism is an example of developmental evolution that includes the colony level, in that the environmental input to a developing larva becomes socially regulated. The evolution of caste dimorphism thus involves changes both in the rearing of larvae and in the developmental response to the rearing. Our aim is to elucidate this coevolutionary process. This entails an identification of the basic properties of the rearing procedure, for instance the ingredients of the larval diet that act as cues for development, and the nature of the developmental response to the rearing.

We approach the question using a mathematical model. Traditionally, ideas about the regulation of development have played significant roles in conceptual treatments of caste polyphenism (Wheeler 1986; West-Eberhard 1996; Linksvayer and Wade 2005; Page and Amdam 2007), but so far, there has been no comprehensive analysis that synthesizes what is known about the developmental evolution of this syndrome. We perform such an analysis for the well-studied case of the honeybee by constructing a model of the rearing and development of queens and workers, based on available information about developmental and behavioral processes, and then comparing the model results with experimental data on the caste morphospace obtained from hive and laboratory rearing of larvae. Among the important components of the model are, first, the implementation of distinct nurse feeding regimes for worker- and queen-destined larvae and, second, the regulation of worker ovary development, and hence worker reproductive potential, by programmed cell death (PCD) of ovarioles (Schmidt Capella and Hartfelder 1998, 2002) as a response to nurse-mediated food restriction.

Ovariole PCD may have been present in some form before the evolution of the honeybee caste dimorphism and might have been co-opted into this developmental system. PCD is a component of the developmental regulation of reproductive investment in many different organisms (Baum et al. 2005). There are also observations of ovariole PCD influencing caste development in stingless bees (Boleli et al. 1999), although this developmental process is probably not homologous to that in honeybees, because it occurs in pupal rather than larval development and results in the complete destruction of the ovaries. One possibility for the evolution of PCD as a way of regulating reproduction is that it was originally a general starvation response, which was exploited by honeybee nurses in order to control ovary development in worker larvae.

The diets of honeybee queen and worker larvae are controlled by the feeding behavior of nurse workers. There are queen–worker differences in the amounts fed (such that queens get more), but also differences in the diet composition. A number of properties of the larval diet have been suggested to influence or determine caste development (Dietz and Haydak 1971; Asencot and Lensky 1976, 1985, 1988; Chittka and Chittka 2010; Kamakura 2011). For our modeling, diet differences that contribute to differential queen–worker development are the most important. So, for instance, the sugar content of the diet is a crucial input from the nurses to the larvae, such that the sugar concentration in the food provided to 1- to 3-day-old worker larvae is considerably lower than that provided to queens, and this is known to influence the developmental trajectory (Asencot and Lensky 1976, 1985, 1988).

As an another possibility, a recent study (Kamakura 2011) showed that royalactin, a major royal jelly protein (MRJP), influences larval growth and development and is needed for the full development of a queen phenotype. Royalactin (also known as monomeric MRJP1) quantitatively affects growth and developmental rates of larvae through activation of the epidermal growth factor (EGF) receptor (Egfr) pathway (Kamakura 2011). However, hive-reared larvae are continuously fed fresh royal jelly (queens) or a mixed diet containing fresh royal jelly (workers), indicating that both queens and workers ingest royalactin. Queen and worker larval diets in fact contain quite similar concentrations of protein (Shuel and Dixon 1959, 1960), with essentially the same complement of MRJPs (Schmitzová et al. 1998). Based on available information, it then seems unlikely that a queen–worker diet difference in the concentration of royalactin is the sole determinant of caste in naturally reared honeybees. Royalactin might still serve as a (redundant) quantitative nutritional signal, but it appears that sugar is a more important differential determinant of the caste dimorphism. For this reason, we have chosen to focus on the sugar concentration in larval food.

Two properties of the feeding regimes have particular significance in the model: a reduced sugar content of the food given to young worker-destined larvae, which lowers their metabolic rate and hemolymph juvenile hormone (JH) titer and induces ovariole PCD; and a reduced amount of food to older worker-destined larvae, which makes them smaller. A striking feature of the evolution of caste dimorphism is that social behavior, in the form of the nurse feeding regimes, has become integrated into a colony-level developmental network that produces the dimorphism (Linksvayer et al. 2011, 2012a,b). As an illustration of the colony-level integration of social behavior and individual development, we find that the discrete queen–worker dimorphism is the result of discrete feeding regimes imposed by nurse workers, whereas a range of artificial feeding regimes result in a range of phenotypes that include queen–worker intercastes.

## Model

The model specifies how larval development and nurse worker feeding behavior together determine the phenotype of an individual (queen or worker) honeybee. The phenotype of an individual is two-dimensional (*x*, *y*), where *x* is the body size (weight) and *y* is the ovary size measured as total number of ovarioles (summing over both ovaries). Additional model details and explanations are presented in the Appendix.

### Feeding regimes and JH profiles

**Figure 2:**
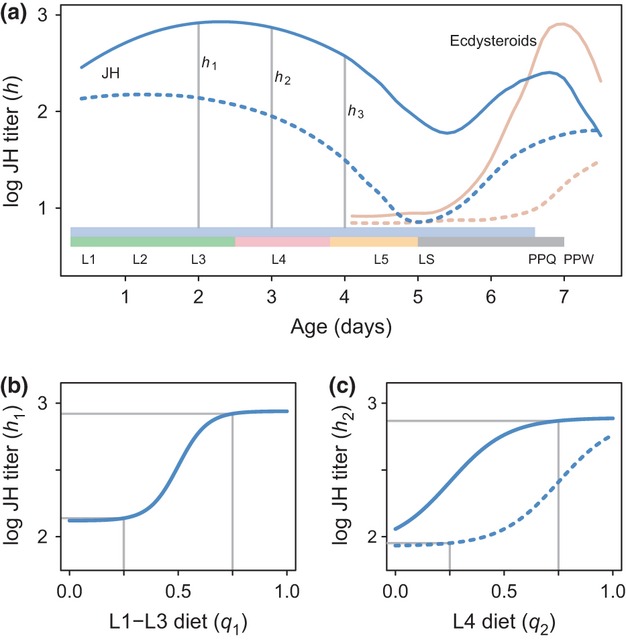
Feeding regimes and hormonal profiles of developing queens and workers. (a) The hemolymph JH titers (pmol/mL) of queens (blue curve) and workers (dashed blue) respond to the feeding regimes imposed by nurses. Queen food is unrestricted and contains about 12% sugar (light blue bar), whereas worker food changes over development (multicolored bar). During the first three instars (L1–L3) worker food is unrestricted, but contains only around 4% sugar (green). Feeding is restricted in the fourth instar (pink) and in the fifth, the sugar content is increased (orange). After nurses seal the worker cells (LS), workers starve (gray) through to the prepupal stage (PPW), whereas queen cells are mass provisioned at sealing, so queens continue feeding until the prepupal stage (PPQ). (b) The L3 JH titer *h*_1_ is a response to diet sugar content (*q*_1_; normalized to a 0–1 range), and influences growth and development. (c) The L4 JH titer *h*_2_ is a response to both L1–L3 and L4 diet (blue curve, *q*_1_ = 0.75; dashed blue, *q*_1_ = 0.25). High JH titers protect ovarioles from PCD, induced when ecdysteroid titers rise to initiate metamorphosis (beige curves in [a]). Based on Asencot and Lensky (1988), Rembold et al. (1980), Rembold (1987), Rachinsky et al. (1990) and Shuel and Dixon (1968), the curves in (a) are LOESS fits to empirical data.

In honeybee queen and worker development, the timing, quality, and amount of food delivered by nurse workers influence the JH profiles of larvae (Fig. 2a), which in turn direct the developmental trajectories of the castes. We treat the first three larval instars as one component or phase, because queens and workers each receive constant feeding schedules during this phase and because the effect of feeding during the first two instars can be overridden in the third instar (Nijhout and Wheeler 1982). Experimental manipulation of larval diet (Asencot and Lensky 1985, 1988) and topical application of JH (Nijhout and Wheeler 1982; Asencot and Lensky 1984; Antonialli and da Cruz-Landim 2009) have established that the sugar content of the food during the third and fourth instars (L3 and L4) influences the hemolymph JH titer, and that this in turn determines queen versus worker development. As illustrated in Figure 2b, we model the influence of L3 diet *q*_1_ on JH as


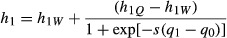
(1)

where *h*_1_ is the base-10 logarithm of the JH titer in L3 (Fig. 2a) and *h*_1*Q*_, *h*_1*W*_, *s,* and *q*_0_ are parameters. In the same way, the JH titer in L4 depends on the L3 and L4 diet (Fig. 2c),


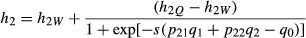
(2)

There is a similar relation for the JH titer *h*_3_ in L5


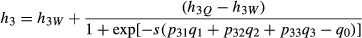
(3)

which contains a number of parameters.

### Reproductive allocation

**Figure 3:**
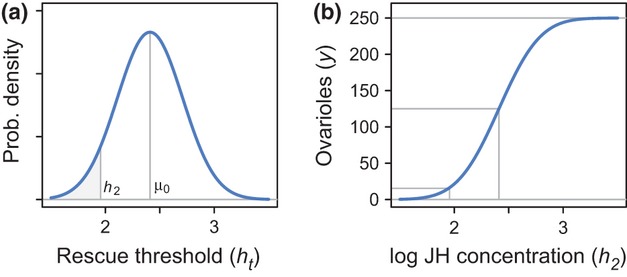
Reproductive allocation, for a case where ovarioles are only available for rescue in L4, so that *r*_2_ = 1 in equation (4). (a) The probability distribution of rescue thresholds: an ovariole is rescued if *h*_2_ > *h*_*t*_. (b) The resulting relation from equation (4). The example illustrated by the shading, indicating that ovarioles with thresholds less than *h*_2_ are rescued, corresponds to a worker allocation of ovarioles. Ovariole number (*y*) is given as a sum of the ovariole numbers of both ovaries.

The JH titer causes developing ovariole primordia to be rescued from PCD (Schmidt Capella and Hartfelder 1998, 2002), in a process spanning L3, L4, and early L5 (Dedej et al. 1998; Antonialli and da Cruz-Landim 2009). We model this process as a distribution of rescue thresholds for ovarioles. In each of the phases, a proportion of the ovarioles are available for rescue by the JH titers *h*_1_, *h*_2_, and *h*_3_, respectively. Let *h*_*t*_ be the rescuing threshold of an ovariole, in the sense that ovariole PCD is prevented if the log JH titer *h* is above the threshold: *h* > *h*_*t*_. We assume that there is random variation in the rescue threshold between ovarioles, such that *h*_*t*_ is normally distributed with mean *μ*_0_ and standard deviation *σ*_0_. A proportion *r*_1_ of the ovarioles are available for rescue by *h*_1_, and similarly the proportions *r*_2_ and *r*_3_ by *h*_2_ and *h*_3_, respectively (*r*_1_ + *r*_2_ + *r*_3_ = 1). Assuming that the distribution of rescue thresholds is the same for the different phases, the number of ovarioles after PCD, as a function of the JH titers, is


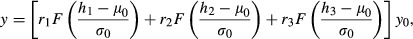
(4)

where *F* is the standard normal cumulative distribution function and *y*_0_ is the number of developing ovarioles present before the onset of PCD. See Figure 3 for an illustration of the reproductive allocation. In this way, the larval development of ovariole primordia and the diet-modulated, and thus nurse-controlled, JH profile together determine the number of ovarioles of the adult insect. Although one might have expected that queen larval development involves laying down more ovariole primordia than worker larval development, this is not the case in honeybees (Hartfelder and Steinbrück 1997), so we ignore this possibility in the model. Apart from ovarioles, there are other important consequences of the JH titer, including higher respiration rates in queen-destined larvae (Shuel and Dixon 1959; Eder et al. 1983), accompanied by higher feeding expectation and higher potential growth rate.

### Critical weight and size determination

**Figure 4:**
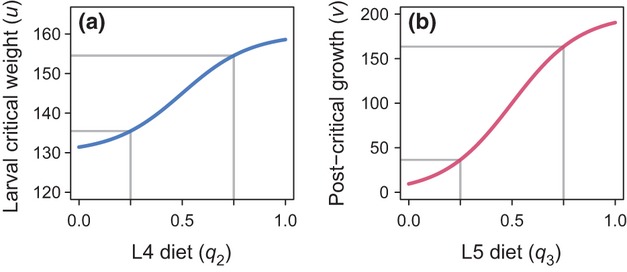
Determination of the larval critical weight and postcritical growth increment. (a) Larval critical weight *u* (mg) as a function of the L4 diet *q*_2_, as given by equation (5). (b) The postcritical growth increment *v* (mg) as a function of the L5 diet *q*_3_, as given by equation (6).

Certain of the mechanistic aspects of larval growth and metamorphosis in holometabolous insects are well established and are thought to hold generally, so they should also apply to honeybees. These include the basic observation that larval growth tends to follow Dyar's rule, stating that the proportional size increase between successive instars is approximately constant, which holds for honeybees (Rembold et al. 1980; Cnaani and Hefetz 2001), as well as the regulatory role of the so-called critical weight (Mirth and Riddiford 2007). In the model, the L4 diet *q*_2_ influences the critical weight *u*


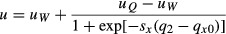
(5)

and the amount *q*_3_ fed during L5 influences the postcritical growth increment *v*


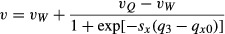
(6)

(these equations are illustrated in Fig. 4), and *u* and *v* together determine the final weight


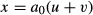
(7)

where the parameter *a*_0_ gives the proportional reduction in weight, from the maximal larval weight to the adult weight at eclosion. In summary, the model of size determination we use is inspired by previous modeling of insect growth (Nijhout et al. 2006, 2010). The L4 feeding determines the critical weight and after critical weight has been reached in L5, there is a more or less fixed time interval in which a larva will continue to feed. The weight increment it achieves in this final period of growth is determined by the quantity of the food it receives.

Finally, in addition to the effects directly represented in the model, it is likely that the target of rapamycin (TOR), Egfr and insulin signaling pathways are involved in the determination of size (Mirth and Riddiford 2007; see also Wheeler et al. 2006; Patel et al. 2007; Kamakura 2011 for honeybees), as well as in honeybee caste determination in general (Wheeler et al. 2006; Patel et al. 2007; Kamakura 2011; Mutti et al. 2011). However, at least for insulin signaling, there is no simple relation with growth rates or molecular markers of oxidative metabolism in queen and worker honeybees (Azevedo and Hartfelder 2008; Azevedo et al. 2011).

## Results

**Figure 5:**
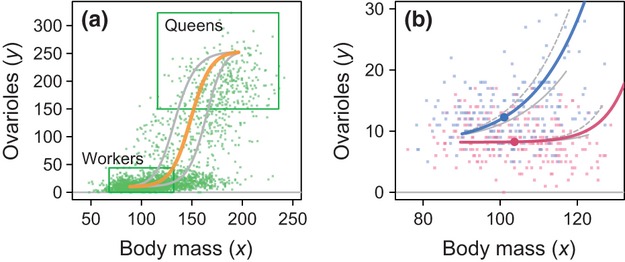
The queen–worker ovary size versus body size spectrum. (a) The cloud of light green points represents observed body weights (mg) and total ovariole counts of individual honeybees from different origins and reared under varied conditions (hive reared, cross-fostered as well as laboratory reared; 3610 individuals (Linksvayer et al. 2011; Page and Fondrk 1995; Kaftanoglu et al. 2010). The cloud maps out a total phenotypic space. The green boxes delineate the phenotypic subspaces of hive-reared individuals, showing that the distinctness of queens and workers is a consequence of distinct feeding regimes imposed by nurses. The model generated, fitted phenotype set (orange curve) illustrates the effect of variation in food quality and quantity (*q*_1_, *q*_2_, and *q*_3_ vary in parallel from 0.1 to 0.9). Two model variants are shown, where larvae either have a stronger (upper gray curve) or weaker (lower gray curve) JH response to the quality and quantity of food. (b) Observed and model-fitted phenotype sets of workers of two strains of honeybees, selected for either high (blue points and curve) or low (red) pollen hoarding behavior (Kaftanoglu et al. 2011; Linksvayer et al. 2011). The model fits entail a less threshold-like JH response to the diet for high-strain bees as compared with low-strain bees. The gray lines show the effects of high- versus low-strain-rearing environments on the model fits (solid gray: reared by high-strain nurses; dashed gray: reared by low-strain nurses).

Data on body mass and the number of ovarioles of honeybees reared under diverse conditions, including artificial and hive rearing, show that these two key caste-dimorphic characters are correlated and vary continuously, forming a single cloud in phenotypic space rather than two distinct clouds (Fig. 5a). Two clouds would be expected if the caste dimorphism arises from a developmental switch intrinsic to a larva. The single cloud indicates that the switch is extrinsic and controlled by the nurses: when nurse bees control the feeding of larvae, two distinct distributions of phenotypes are observed (the boxes in Fig. 5a). The model output spans the observed phenotype space, allowing for variation in the quality and quantity of feeding and variation in model parameters (Fig. 5a).

**Figure 6:**
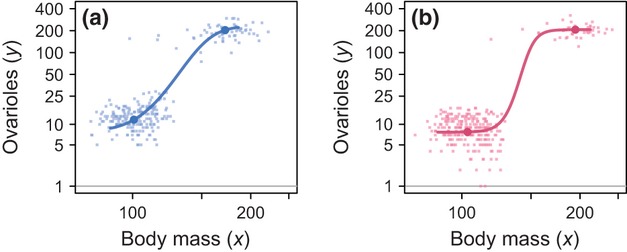
Observed and model-fitted phenotypes of queens and workers of two strains of honeybees, selected for either (a) high or (b) low pollen hoarding behavior (Linksvayer et al. 2011). Body weights and ovariole numbers are shown on logarithmic scales. The worker data and model fit (lower left cloud in (a) and (b)) are the same as those shown in Figure 5b.

The relationship between body mass and ovariole number, however, is not fixed, but can differ among genotypes of honeybees and is selectable (Figs. 5b, 6). For the high and low pollen hoarding strains (Page and Fondrk 1995), selection for more stored pollen resulted in worker bees with a greater tendency to collect pollen and more ovarioles. From the model fitting (details in the Appendix), the higher number of ovarioles of high-strain workers, as well as the higher body weight–ovariole number correlation for these workers (Figs. 5b, 6), is explained by a higher (Amdam et al. 2010). Based on the model fitting, this difference in JH response to diet is statistically significant (see Appendix). Cross-fostering and laboratory-rearing studies have previously shown that larvae of the two strains respond differently to nutritional inputs with regard to the relationship between ovariole number and body size, as well as to other queen–worker dimorphic traits (Linksvayer et al. 2011).

**Figure 7:**
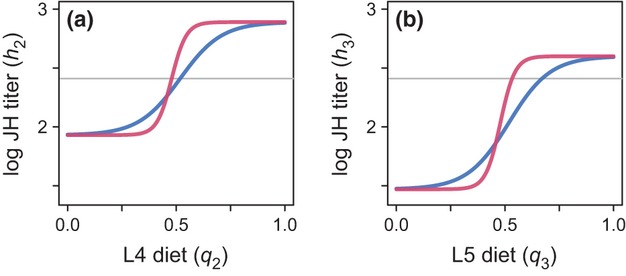
Model fitted JH titers as a function of diet for high- (blue) and low-strain (red) bees in the middle (a) and late (b) phases of the feeding regime. The horizontal gray line indicates the mean ovariole rescue threshold *μ*_0_.

In addition to the difference between strains in developmental responses to feeding, differences in the feeding regimes of nurses have been inferred (Linksvayer et al. 2011). Our model fitting indicates that the quality/quantity of the L4 worker diet supplied by low-strain workers is higher than that supplied by high-strain workers (this difference is statistically significant; see Appendix). In conjunction with the differences in developmental responses between high- and low-strain larvae, the effect is that the ovariole numbers of high-strain workers respond more strongly than low-strain workers to this larval diet variation, as illustrated in Fig. 5b (dashed and solid gray lines). This result is consistent with the findings of Linksvayer et al. (2011).

As seen in Figure 2a, the queen feeding regime is relatively simple, with ad libitum feeding of secretions from nurse workers' hypopharyngeal glands throughout the larval development. Workers, on the other hand, require a more complicated feeding program that includes phases with lower sugar content and restricted amounts. The more complex nutritional program of the worker larva, compared with the queen larva, provides a clue to the evolutionary history of the queen and worker phenotypes and suggests the scenario in Table 1. In the scenario, larval and nurse bee control of caste development evolve together, such that larvae evolve to respond appropriately to the nutritional inputs of the nurse bees, and nurse bees evolve the appropriate feeding behavior and glandular nutritional components to shape the queen and worker phenotypes. This joint evolution of the developmental program of caste differentiation is a signature of colony-level selection and gives rise to a superorganism (Hölldobler and Wilson 2009; Linksvayer et al. 2011).

**Table 1:** Evolutionary history of honeybee development and feeding regimes

Stage	Developmental state or change	Feeding regime
0	Ancestral nutrition-related ovariole length – body size allometry; ancestral ovariole number (total 8)	Ancestral seasonal variation in feeding of larvae, with more workers per larva, and thus more food, toward the end of the season; mass provisioning with all food of similar, high quality
1	Same nutrition-related ovariole length and body size variation as before, but a greater amplitude (bigger “queens”)	Simultaneous differential feeding of individuals, with more food to “queens” and restricted food to “workers” during the last larval instar; the increased caste dimorphism is favored by larger colony size
2	Increased ovariole numbers, favored by larger colony size in combination with swarm founding by old queens; ovariole primordia develop early and there is nutrition- and JH-mediated ovariole PCD (in parallel, male testiole numbers also increase)	Same differential feeding as before
3	Increased amplitude in JH-mediated ovariole number variation, providing the colony advantage of the L1-L4 diet manipulation	Worker-destined larvae are fed less in L4 and the sugar content of the L1-L4 worker diet is lowered; no change in L5 sugar content, which is needed for metamorphosis (Shuel and Dixon 1968)
4	Divergence of other queen and worker traits, as signaled by the differential feeding regimes	Queen feeding regime essentially the ancestral one; honeybee worker feeding regime now in place
5	Extant honeybee development	Extant honeybee queen and worker feeding regimes

The scenario starts form a primitively eusocial ancestral state (Kawakita et al. 2008; Cardinal and Danforth 2011) and proceeds to the current honeybee state. In each of the stages 1–4, there is evolutionary change in the larval development and/or the nurse feeding regimes.

## Discussion

The overall purpose of the model is to integrate current knowledge about the regulation of caste-dimorphic development in honeybees, providing sufficient detail to enable a fit of the model to data on realized phenotypes. In particular, the model gives an explanation for the observed correlation of body weight and ovariole number: the correlation derives from a partial overlap and correlation of the feeding-regime-mediated inputs to the determination of body weight and ovariole number of developing larvae. Other caste-dimorphic characters such as mandible shape, development of the corbicula and pollen brush (pollen collecting apparatus of workers), and wax mirrors (part of the wax producing glands of workers) also vary continuously, are determined at different stages of development, and correlate to a greater or lesser degree with body weight and ovariole number (Dedej et al. 1998; Linksvayer et al. 2011).

The model allows us to pinpoint the differences in the developmental responses of body weight and ovariole number to diet between high- and low-strain bees (Figs. 5b, 6, 7), as well as the differences between the feeding regimes of high- and low-strain nurses. These differences are the result of selection on a colony-level trait, the amount of stored pollen (Page and Fondrk 1995), illustrating the integration of colony-level processes and individual larval development (Linksvayer et al. 2009, 2011, 2012a,b). According to our analysis here, the high-strain worker larvae have, by way of a higher and more diet-responsive JH titer, become modified to rescue a higher proportion of their ovarioles, but at the same time, the high-strain nurses have lowered the quality/quantity of the diet provided to L4 worker larvae, such that, to a degree, the diet change counteracts the increase in ovariole number. The net result is that adult high-strain workers have somewhat more ovarioles and a slightly lower body weight compared with low-strain workers (Fig. 5b).

The representation of the developmental mechanisms in the model provides a conceptual framework for evolutionary scenarios such as the one shown in Table 1, entailing a joint and successive evolution of the model-represented components of the developmental program. The scenario in Table 1 is not intended to suggest that there is a single evolutionary route or series of steps toward high social complexity in bees. In fact, another group of corbiculate bees, the stingless bees (Meliponini), has reached similar levels of social complexity, also involving larval provisions, body size diphenism, and caste differences in the JH titer (Hartfelder and Rembold 1991), but without caste differences in ovariole number. This difference implies an early branching in corbiculate bee social evolution after stage 1 of Table 1, with Apini on one branch and Bombini and Meliponini on the other (Kawakita et al. 2008; Cardinal and Danforth 2011). Such alternative routes are consistent with the distinct forms of swarm founding in Apini and Meliponini, which have a relation to a parallel versus serial organization of the egg maturation in queens, in the form of more but shorter versus fewer but longer ovarioles. In Apini, old queens establish colonies by swarming and can benefit from many ovarioles and a correspondingly shorter abdomen, by combining a high egg-laying capacity with efficient flight (stage 2 of Table 1), whereas in Meliponini young queens establish colonies by swarming, at an age before their ovarioles have been activated. Mature, egg-laying queens of stingless bees are incapable of flying, partly because their abdomens are greatly distended, and they are referred to as physogastric queens.

From the perspective presented here, a honeybee colony, just as the colonies of other social insects, acts as a regulatory network, with both development and behavior associated with differential gene expression profiles (Evans and Wheeler 2000). Gene batteries are the ultimate targets of such regulatory states, and in honeybee caste development, these are not only composed of *cis*–*trans* regulatory networks (Evans and Wheeler 2000; Cristino et al. 2006) but also involve extensive epigenetic modification (Kucharski et al. 2008). Moreover, signals that activate or deactivate gene batteries are coming not only from cells within an organism, but are also the result of the behavioral interactions between the developing larvae, the nurse worker bees, and the queen. One signaling mechanism involves nutrition – the timing, amount, and quality of food. The colony-level social network is part of an extended regulatory network, where nurse behavior influences the development of individual larval phenotypes. The colony is then a superorganism (Hölldobler and Wilson 2009), in the sense of a developmental unit, for which the regulation of the caste dimorphism is a primary task.

## Appendix: Model Details

We refer to the larval developmental machinery as a “module,” in the sense of an integrated functional unit, and to nurse behavior as another module. Each module has a number of characteristics, which are implemented as model parameters and can be modified in evolution.

### Feeding regimes and JH titer profiles

The feeding regimes we consider are illustrated in Figure 2. There are three phases: early (L1–L3), middle (L4), and late (L5). For each of these phases, we assume that there is a food quality/quantity variable that is controlled by the nurse workers. Thus, we have the inputs *q*_1_, *q*_2_, and *q*_3_, each of which we allow to vary between 0 and 1. For the first phase, we assume that the relevant aspect of the food is the sugar content (there are no restrictions on the amount of food during this phase). We let the 0–1 range of food quality *q*_1_ correspond to the range from 0% to 16% sugar, so that larval jelly (4%) has *q*_1_ = 0.25 and queen jelly (12%) has *q*_1_ = 0.75 (Shuel and Dixon 1968; Asencot and Lensky 1988).

To summarize the feeding regimes (Fig. 2), queen and worker larvae are fed basically the same food ad libitum for the first 2.5 days of life (L1–L3), except queens get around 12% sugar in their food compared with about 4% for worker-destined larvae. Worker larvae are food restricted in L4, but at the start of L5, the sugar concentration is increased to about that of queen larvae. This is necessary, for otherwise the larvae are unable to pupate and will die (Shuel and Dixon 1968). Queen larvae are sealed in their cells about 5 days after hatching from the egg and have a surplus of food on which they feed for an additional 1–1.5 days. Worker larvae are sealed in their cells with no surplus of food.

The queen developmental trajectory is associated with a higher JH hemolymph titer and higher metabolic rate. On the basis of a dose–response experiment (see Fig. 5 in Asencot and Lensky 1984), we use the logarithm of the JH hemolymph titer and denote its value by *h*. The logarithm of the JH concentration in mid-L3 is denoted by *h*_1_ and is a sigmoid function of the food quality *q*_1_, as shown by equation (1) in the main text. The parameters *h*_1*Q*_ and *h*_1*W*_ in this equation are the queen and worker log JH titer asymptotes of the larval module and *s* and *q*_0_ are further larval module parameters, expressing the steepness and inflexion point of the log JH response to food quality (see Fig. 2a and b). The logarithm of the JH concentration in mid-L4 is denoted by *h*_2_ and is a sigmoid function of the food quality/quantity inputs *q*_1_ and *q*_2_, as shown by equation (2), where *h*_2*Q*_ and *h*_2*W*_ are queen and worker log JH titer asymptotes of the larval module and *p*_21_, *p*_22_ = 1 − *p*_21_ are larval module parameters, giving the relative influence of the previous and current feeding on JH (see Fig. 2a and c). Similarly, for the early L5 log JH titer, the dependence on feeding is given by equation (3), which contains additional larval module parameters.

### Reproductive allocation

We assume the same number *y*_0_ of ovariole primordia, regardless of L1–L3 and L4 feeding regimes, which is consistent with the observation (Hartfelder and Steinbrück 1997). As a consequence of the influences from L3 up to the start of and including the early part of L5, the larval module determines the reproductive allocation (Dedej et al. 1998; Antonialli and da Cruz-Landim 2009). The mechanism depends on a diet-modulated JH concentration that rescues ovarioles from programmed cell death (PCD; Schmidt Capella and Hartfelder 1998, 2002; Antonialli and da Cruz-Landim 2009). In the model, we assume that a certain proportion of the ovarioles are available for rescue by each of the JH titers *h*_1_, *h*_2_, and *h*_3_ (Fig. 2), as described by equation (4) and illustrated in Figure 3. We can think of *y*_0_, *r*_*i*_, *μ*_0_, and *σ*_0_ in equation (4) as parameters that are set in the larval module.

### Larval critical weight

Critical weight is the size in the final larval instar at which JH secretion by the corpora allata stops, and the subsequent reduction in JH titer acts as a starting signal for a sequence of events that eventually result in metamorphosis (Nijhout et al. 2006, 2010; Mirth and Riddiford 2007; Mirth et al. 2009). Feeding and growth do not cease immediately at the critical weight, so that the insect can become considerably larger than the critical weight, but the critical weight is a basic component of the system that regulates insect growth. The critical weight can be defined as the size beyond which starvation no longer can delay metamorphosis (Mirth and Riddiford 2007; in *Manduca*, starvation after the critical weight is reached does not change the timing of metamorphosis, but in *Drosophila,* such starvation instead shortens the time until metamorphosis).

The critical weight of a given individual depends on its weight at the start of the final instar and, possibly, its growth rate in previous instars (Nijhout et al. 2006, 2010), and will therefore be influenced by things like diet quality and JH titers in the instars prior to the final larval instar. The critical weight should thus be set (but not yet reached) at the start of the final larval instar, and can depend on aspects of the previous growth.

In our case, the critical weight can be a function of the feeding regime up to and including L4, which could mean that it is influenced by both *q*_1_ and *q*_2_. However, a higher growth rate of queens compared with workers emerges only in L4 or early L5; in late L3, worker-destined larvae instead have a higher growth rate (Stabe 1930; Weiss 1974; Asencot and Lensky 1985). For this reason, we assume that the critical weight *u* depends only on *q*_2_, according to equation (5) in the main text, which represents a sigmoid function where *u*_*Q*_ and *u*_*W*_ are the queen and worker asymptotic critical sizes of the larval module, and *s*_*x*_ and *q*_*x*0_ are other larval module parameters (the assumption only holds approximately: experimental grafting of differently aged worker larvae into queen cells show a small effect of late L3 diet on final body weight, with around 10% smaller body weight for late L3 worker diet compared with queen diet (Weiss 1974), indicating a certain influence of late L3 diet on critical weight). See Figure 4a for an illustration of the setting of the critical weight.

### Size determination

In the L5 feeding regime, there is restricted food for workers and ad libitum food for queens. Workers also receive a high sugar concentration in L5, which is needed for metamorphosis (Shuel and Dixon 1968), so *q*_3_ measures the amount of food during L5. The amount of food received and the onset of the starvation regime for workers in L5 thus regulates the postcritical growth increment *v*. We express this in equation (6) in the main text, where *v*_*Q*_, *v*_*W*_, *s*_*x*_, and *q*_*x*0_ are larval module parameters. See Figure 4b for an illustration. The final weight *x* is now given by equation (7), where the parameter *a*_0_ is the proportional reduction in weight, from the maximal larval weight to the adult weight at eclosion.

### Fitting the Model to Data

The model is expressed in equations (1)–(7), which contain a number of parameters. Although the feeding regimes and larval development of worker and queen honeybees have been studied in considerable detail over a period of many decades, there is still limited information about several of the processes that are represented in the model. For instance, as illustrated in Figure 2a, there are data on log JH titer profiles of worker and queen larvae, but there is no detailed information on how the quality and quantity of larval food during different phases of development influence these profiles. There are also no studies on the detailed mechanisms of honeybee larval growth, involving concepts such as critical weight that have been developed in the studies of *Manduca* and *Drosophila*. For these reasons, our general approach is to first set a number of parameters to be broadly consistent with available information about queen and worker growth and development, and then to use statistical Markov Chain Monte Carlo (MCMC) modeling to fit the rest of the parameters to data from the hive rearing of high- and low-strain bees in Linksvayer et al. (2011). The reason it is not possible to fit all the model parameters using the high- and low-strain data is that the data consist of body weights and ovariole numbers, which in themselves are not enough to fully determine all parameters. A summary of the choosing and statistical fitting of parameter values appear in Table A1.

As a starting point, we assume that the average food quality/quantity to be *q*_1_ = *q*_2_ = *q*_3_ = 0.25 for workers and *q*_1_ = *q*_2_ = *q*_3_ = 0.75 for queens. This can be seen as a normalization of the variation in feeding regimes between workers and queens. Given this assumption, there is a good fit to the values for workers and queens of the log JH profiles in Figure 2a (*h*_1_, *h*_2_, and *h*_3_) when using the parameter values for *h*_1*W*_, *h*_1*Q*_, *h*_2*W*_, *h*_2*Q*_, *h*_3*W*_, and *h*_3*Q*_ appearing in Table A1, together with an assumption of *s* = 15 and *q*_0_ = 0.5, in equations (1), (2), and (3). The values of *p*_21_, *p*_31_, and *p*_32_ do not matter for this fit, but where needed, we used the values shown in Table A1, which are reasonable, but otherwise not known. In summary, for equations (1), (2), and (3), we assumed reasonable values for the asymptotes and the parameters *p*_*ij*_, and we use MCMC model fitting of *s* and *q*_0_ to high- and low-strain bees (see below and Table A1). These latter two parameters allow some, but of course not total, freedom in describing different shapes of the dependence of the log JH titer on food quality/quantity. In Figure 2b and c, the parameter values are as shown in Table A1, except that *s* = 15 and *q*_0_ = 0.5 were used in the figure.

Concerning the parameters in equation (4), data from Dedej et al. (1998) and Antonialli and da Cruz-Landim (2009) give an indication of the ability of an increased JH titer to rescue ovarioles at different points in larval development. On the basis of these data, we have the values of *r*_1_, *r*_2_, and *r*_3_ in Table A1 as a rough approximation (about half of the ovarioles can be rescued by a high JH titer in the final instar). The values for *μ*_0_ and *σ*_0_ should be such that there is a shift from worker to queen allocation of ovarioles as the log JH titer varies over its observed range, and we used MCMC model fitting to obtain the estimates in Table A1 (these estimates were also used in Fig. 3). The number *y*_0_ of ovariole primordia for high- and low-strain bees was estimated through MCMC model fitting (see below and Table A1).

As mentioned above, the critical weights of honeybee workers and queens have not been determined experimentally, but are likely to be reached somewhat before the sealing of cells, because the worker feeding regime includes starvation after sealing. This suggests that workers grow rather little after reaching critical weight, whereas queen larvae may gain more than half of their maximum body weight through postcritical growth. As a reasonable approximation for parameters in equations (5) and (6), we used the values of *v*_*W*_, *s*_*x*_*,* and *q*_*x*0_ given in Table A1. The value *v*_*W*_ = 0 expresses that starvation, after the critical weight is reached, results in completion of development without additional growth. For the illustration in Figure 4, we used *u*_*Q*_ = 160, *u*_*W*_ = 130, and *v*_*Q*_ = 200 (see below and Table A1 for MCMC model fitting of these parameters to high- and low-strain bees). The parameter values for equations (5) and (6) entail that most of the weight difference between queens and workers is a result of postcritical growth (Fig. 4), which is in broad agreement with effects of late (L5) starvation of queen-destined larvae. Such starvation can result in fully developed queens emerging with body weights similar to those of normal workers. Finally, for equation (7), we used the maximal larval body weights of queens and workers from the growth curves in Stabe (1930) and Weiss (1974), together with weights of newly emerged adults from Linksvayer et al. (2011), to estimate *a*_0_ as specified in Table A1.

#### Fitting to high- and low-strain bees

In order to investigate how well our model can describe the observed relation between ovarioles and body weights for high and low pollen hoarding strains (Linksvayer et al. 2011), we implemented the model in the JAGS/BUGS statistical model fitting language and estimated parameters from MCMC runs, using the “rjags” package (Plummer 2011) for the R statistical software (R Development Core Team 2011). The advantage of this approach, compared to more standard statistical modeling such as linear regression or mixed models, is that it is possible to tailor the form of the dependence between independent variables with greater flexibility. In our model, the independent variables are the feeding regimes and the dependent variables are body sizes and ovariole numbers. The model connects these through a series of equations, corresponding to equations (1)–(7), which can be expressed in the JAGS/BUGS model fitting language. By selecting reasonable prior distributions for the model parameters and running the MCMC simulations, one obtains estimates of the parameters in the form of posterior means, together with Bayesian confidence intervals.

The outcome of this model fitting procedure is illustrated in Figures 5b, 6. The major difference between the fitted parameters for high- and low-strain bees is illustrated in Figure 7; the two strains have contrasting JH profiles as a function of diet. The high-strain bees have a less threshold-like, and thus more gradually increasing profile, entailing higher JH titers for high- compared with low-strain worker larvae (Fig. 7). This difference is statistically significant, in the sense that the 95% Bayesian confidence intervals for the slopes *s*_*H*_ and *s*_*L*_ in Table A1 are nonoverlapping. For the other parameters in equations (1)–(6) that were estimated through MCMC model fitting, the Bayesian 95% confidence intervals overlapped substantially between high- and low-strain parameters. This indicates that it is uncertain whether high- and low-strain larval developmental responses differ in ways other than in the JH titer response to variation in diet.

The details of the model fitting are as follows: to achieve reasonably homogeneous variation around an average relation between body weight and ovarioles, we log transformed the data from the high- and low-strain hive rearing in Linksvayer et al. (2011), as illustrated in Figure 6. In addition to the model components given by equations (1)–(7), we allowed for normally distributed random variation in the log-transformed body weights and ovarioles, with separate variances for worker- and queen-rearing conditions, expressed as the standard deviation parameters *σ*_*xW*_, *σ*_*xQ*_, *σ*_*yW*_*,* and *σ*_*yQ*_ in Table A1. We also allowed for random variation in the diets experienced by individual queens and workers, around the mean values of *q*_1_ = *q*_2_ = *q*_3_ = 0.25 for workers and *q*_1_ = *q*_2_ = *q*_3_ = 0.75 for queens, expressed as the standard deviation parameters *σ*_*qW*_ and *σ*_*qQ*_ in Table A1. The idea behind these kinds of variation is to describe variation in phenotypes caused by, on one hand, various forms of developmental noise at the individual level and, on the other hand, variation in the feeding of individual larvae by nurse workers.

In a separate MCMC model fitting, we estimated feeding deviations by high- and low-strain nurses, in the form of the deviations *Δq*_2*W*_ and *Δq*_3*W*_ from the mean values of *q*_2_ = 0.25 and *q*_3_ = 0.25 for workers, and the deviation *Δq*_*Q*_ from the mean values of *q*_*i*_ = 0.75 for queens (see Table A1 for these estimates). According to the model fitting, the most important difference between the strains is that the food quality/quantity *q*_2_ given to workers was lower for high-strain nurses than for low-strain nurses, and this difference was statistically significant, in the sense that the 95% Bayesian confidence intervals for the deviations *Δq*_2*WH*_ and *Δq*_2*WL*_ did not overlap. For the other feeding deviations, the Bayesian 95% confidence intervals overlapped substantially between high- and low-strain bees.

Note finally that the high- and low-strain panels in Figure 1 of Linksvayer et al. (2011) were accidentally reversed (Linksvayer et al. 2012a), which needs to be taken into account when comparing Figures 5b, 6 here with Figure 1 in that article.

**Table A1:** Parameter values

Parameters	Values	Equations	Method of estimation
	2.12, 1.93, 1.47	(1), (2), (3)	Data in Fig. 1
	2.94, 2.89, 2.60	(1), (2), (3)	Data in Fig. 1
	0.5, 0.5	(2)	Reasonable values
	0.25, 0.50, 0.25	(3)	Reasonable values
	0.05, 0.45, 0.5	(4)	Dedej et al. (1998)
	0.0, 6.0, 0.5	(5), (6)	Reasonable values
*a*_0_	0.60	(7)	Stabe (1930), Weiss(1974)
	10.48, 29.62	(1), (2), (3)	MCMC
	0.518, 0.479	(1), (2), (3)	MCMC
	2.41, 0.30	(4)	MCMC
	265.8, 245.3	(4)	MCMC
	134.6, 129.6	(5)	MCMC
	136.4, 159.9	(5)	MCMC
	185.3, 209.1	(6)	MCMC
	0.024, 0.100	–	MCMC
	0.10, 0.12	–	MCMC
	0.42, 0.19	–	MCMC
	−0.072, 0.052	–	MCMC
	−0.002, 0.002	–	MCMC
	−0.012, 0.019	–	MCMC

Versions of the parameters for high and low strain bees are indicated with subscripts H and L. Apart from the parameters appearing in the equations, the table contains estimates of variance components and feeding quality/quantity deviations (see text for further explanation).
